# Embroidery Triboelectric Nanogenerator for Energy Harvesting

**DOI:** 10.3390/s24123782

**Published:** 2024-06-11

**Authors:** Hasan Riaz Tahir, Benny Malengier, Sanaul Sujan, Lieva Van Langenhove

**Affiliations:** Centre for Textile Science and Engineering, Department of Materials, Textiles and Chemical Engineering (MATCH), Ghent University, Technologie park 70A, 9052 Ghent, Belgium; benny.malengier@ugent.be (B.M.); lieva.vanlangenhove@ugent.be (L.V.L.)

**Keywords:** ETENGs (embroidered triboelectric nanogenerators), energy harvesting, wearable electronics, tapping and sliding devices, embroidered stitch length, energy extraction, electrostatic characterization device

## Abstract

Triboelectric nanogenerators (TENGs) are devices that efficiently transform mechanical energy into electrical energy by utilizing the triboelectric effect and electrostatic induction. Embroidery triboelectric nanogenerators (ETENGs) offer a distinct prospect to incorporate energy harvesting capabilities into textile-based products. This research work introduces an embroidered triboelectric nanogenerator that is made using polyester and nylon 66 yarn. The ETENG is developed by using different embroidery parameters and its characteristics are obtained using a specialized tapping and friction device. Nine ETENGs were made, each with different stitch lengths and line spacings for the polyester yarn. Friction and tapping tests were performed to assess the electrical outputs, which included measurements of short circuit current, open circuit voltage, and capacitor charging. One sample wearable embroidered energy harvester collected 307.5 μJ (24.8 V) of energy under a 1.5 Hz sliding motion over 300 s and 72 μJ (12 V) of energy through human walking over 120 s. Another ETENG sample generated 4.5 μJ (3 V) into a 1 μF capacitor using a tapping device with a 2 Hz frequency and a 50 mm separation distance over a duration of 520 s. Measurement of the current was also performed at different pressures to check the effect of pressure and validate the different options of the triboelectric/electrostatic characterization device. In summary, this research explains the influence of embroidery parameters on the performance of ETENG (Embroidery Triboelectric Nanogenerator) and provides valuable information for energy harvesting applications.

## 1. Introduction

Triboelectric nanogenerators (TENGs) are highly promising devices for energy harvesting. They can efficiently transform mechanical energy into electrical energy by utilizing the triboelectric phenomenon and electrostatic induction [1,2,3,4]. It usually involves two materials with contrasting electrostatic characteristics that make contact and then separate, creating an uneven distribution of charges that can be collected as electricity [1]. TENGs have attracted considerable interest for their potential use in wearable electronics, self-powered sensors, and energy harvesting systems. These devices show potential for harnessing energy from different mechanical movements in our everyday activities, such as walking, tapping, or even vibrations. They provide a sustainable option for supplying power to small-scale electronics without depending on conventional energy sources [5,6,7].

Embroidery triboelectric nanogenerators are a versatile method of incorporating energy-collecting abilities into textile-based materials. ETENGs utilize conductive and non-conductive threads and materials with varying triboelectric characteristics to generate energy through mechanical movements and deformation of the fabric [8]. ETENGs provide a smooth integration of energy harvesting capabilities into regular clothing, enabling the development of wearable devices that can generate their own power while being worn. This reduces the need for external power sources and improves the independence and flexibility of wearable technology [9]. ETENGs can combine traditional artistry with advanced nanotechnology. By skillfully combining conductive threads and sophisticated embroidery techniques, these generators readily incorporate energy harvesting capabilities into the structure of fabrics [10]. Embroidery’s adaptability enables the production of intricate patterns and designs that increase both the visual attractiveness of the fabric and the triboelectric performance of the device. ETENGs utilize tactically positioned conductive elements in embroideries to capture mechanical energy generated by different actions, such as tapping, friction, bending, stretching, or airflow. This process effectively converts the clothing into a self-sustaining power generator [11,12].

An important benefit of ETENGs is their compatibility with current textile manufacturing methods, which allows for easy expansion and adaptation for large-scale production. ETENGs, unlike traditional energy harvesting technologies, do not need bulky components or rigid substrates. They provide a lightweight and flexible solution that can easily be integrated into clothing and other textile goods. The intrinsic versatility of this material allows for the creation of a diverse array of applications, such as self-powered wearable electronics used for healthcare monitoring and fitness tracking, as well as energy-generating smart fabrics used in outdoor garments [13]. This technology enables us to utilize ambient energy sources and decreases our dependence on conventional power sources. Embroidery triboelectric nanogenerators have the potential to significantly impact the development of wearable technologies and sustainable energy harvesting [10].

The efficiency of a TENG, such as embroidered triboelectric nanogenerators, is affected by different factors that control the production and accumulation of electrical charges. The selection of materials employed in the device’s construction has a substantial impact on its triboelectric capabilities. Materials with higher surface energies and electron affinities are favored in order to optimize the transfer of charge during contact and separation. Moreover, the physical structure and unevenness of the materials are essential factors in improving the triboelectric effect by increasing the actual contact area and facilitating more effective transmission of electric charge. When it comes to ETENGs, the choice of conductive threads and embroidery techniques has an impact on the overall performance. This is because some thread compositions and embroidery patterns can demonstrate superior triboelectric behavior compared to others [13,14].

Several key variables can enhance the energy-harvesting efficiency of triboelectric textiles. Choosing a triboelectric material with an opposite polarity to improve charge separation during contact and separation movements is essential for optimization [15,16,17]. Additionally, the influence of triboelectric coefficients on the choice of materials for enhancing charge generation should be given substantial consideration [18,19]. Temperature plays a crucial role in determining the electrical output of textile-based triboelectric nanogenerators. Naturally, the performance significantly declines when the temperature is increased from −20 to 150 °C. At temperatures above 260 K, the output of the TENG reduces dramatically because the temperature impacts the capacity of electron energy storage during triboelectrification [20,21]. During contact–separation actions, there is also heat exchange between the surface layers. The thickness of the triboelectric layer directly impacts the device’s capacity to retain electric charge. Experimental evidence has proven that a Triboelectric Nanogenerator (TENG) with a thinner dielectric film can generate voltage, charge, and energy up to 2.5 times more efficiently than a TENG with a thicker layer [22,23]. The manipulation of the surface shape and roughness through treatments, coatings, or nanostructuring has the ability to increase friction and optimize the efficiency of charge generation [24,25]. An increase in the pressure applied to the triboelectric textiles and the size of the surface in contact with the external environment will also improve the transfer of electric charge and, consequently, the efficiency of energy harvesting [20,26,27].

Furthermore, the output performance of the TENG is directly influenced by its mechanical design and setup. The shape and size of the device, along with the mechanical force used during operation, can greatly affect the amplitude and frequency of the electrical signal produced [14,16,17]. When discussing ETENGs, it is important to carefully evaluate the structural integrity and flexibility of the fabric substrate. These factors play a crucial role in determining the device’s capacity to endure deformation and mechanical stress. In addition, environmental conditions such as humidity, temperature, and pressure can impact the triboelectric charging process and thus change the output properties of the TENG [20,28,29]. Comprehending and enhancing these factors are crucial for achieving the highest possible energy conversion efficiency and dependability of triboelectric nanogenerators, particularly in the realm of embroidery-based applications where the design considerations must be precisely balanced with functional performance [30].

In recent decades, there has been significant interest in converting ambient energy into electricity as part of the pursuit of renewable resources and environmental conservation. Various forms of energy can coexist in our surroundings. Industrial and domestic electricity is generated using large-scale electrical energy production equipment like solar power, wind power, hydropower, and geothermal generators [31]. Triboelectric nanogenerators integrated into wearables have the capability to provide electricity for wearable devices and emergency situations as part of the Internet of Things (IoT) [32,33,34]. Triboelectricity and electrostatic charges are frequent occurrences in our everyday lives [35,36]. It is considered to have a dangerous impact due to its potential to cause mishaps through the generation of electrostatic sparks [37,38,39]. The triboelectric nanogenerator idea, on the other hand, reveals a vast source of energy from human biomechanics. The human body is a source of various levels of kinetic action because of its intricate structure and biomechanics [40].

**Figure 1:**
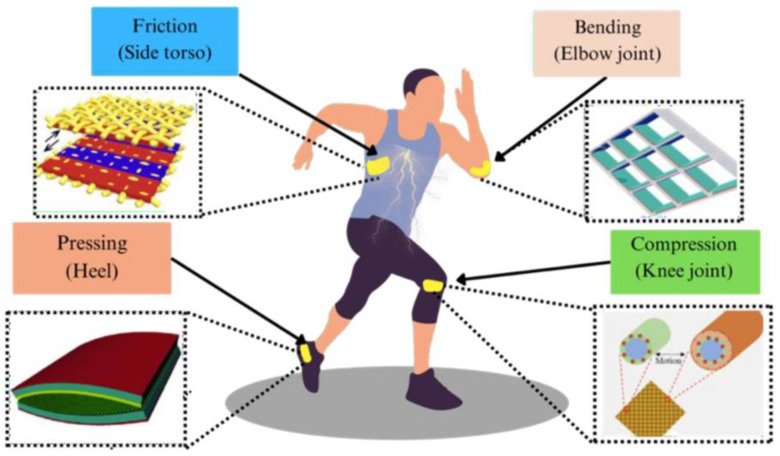
Biomechanical power sources for wearable energy harvesting generators involve techniques such as bending of the elbow, compressing of the knee, pressing of the heel, and generating friction on the side torso.

Due to their high energy conversion efficiency, uncomplicated form, and ease of scaling up, TENGs are ideal for converting human motion into electricity [41,42]. Intermittent low-frequency inputs can effectively serve as a source of energy harvesting while minimizing disruption to the user. Integrating energy harvesters in garments, shoes, braces, and side bags allows for a wide range of adaptable applications [43]. Their reliance can be determined by the substance utilized for construction and its application on human bodily components. Due to their good energy conversion efficiency, simple structure, and scalability, TENGs are ideal for transforming human motion into electricity. They can efficiently harvest energy from irregular low-frequency inputs [44,45,46,47]. Figure 1 shows the biomechanical power sources used by wearable energy harvesting generators. These sources employ several strategies such as elbow bending, knee compression, heel pushing, and friction generation on the side torso.

**Figure 2:**
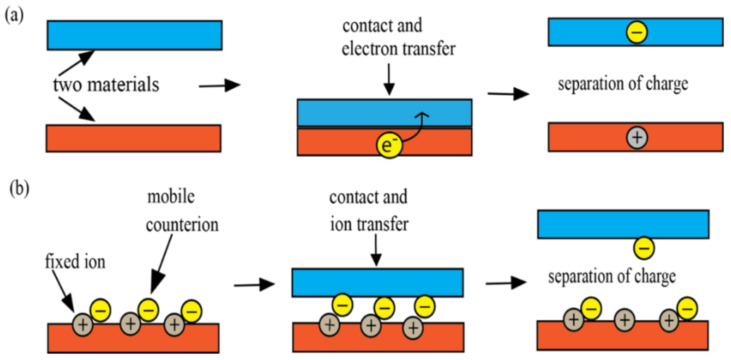
Schematic diagrams of charge transfer mechanisms. (**a**) Electron transfer mechanism; (**b**) ion transfer mechanism.

When two materials come into contact and then separate, it causes the triboelectric effect, also known as triboelectrification or contact electrification, which results in an exchange of electric charge [48]. The first material becomes positively charged, while the second material becomes negatively charged. Friction can amplify the effect, although it is not essential for this occurrence to happen. The triboelectric effect is responsible for the majority of the static electricity we experience in our daily lives [48,49]. Triboelectrification is a phenomenon that results in the generation of opposite charges on the surfaces of materials. The phenomenon can occur in both conductive and insulating materials; however, it is predominantly observed in insulators like rubber, nylon, and PTFE (Teflon) [50,51]. Figure 2 shows schematic diagrams showing the mechanisms of charge transfer, including the electron transfer mechanism and the ion transfer mechanism.

**Figure 3:**
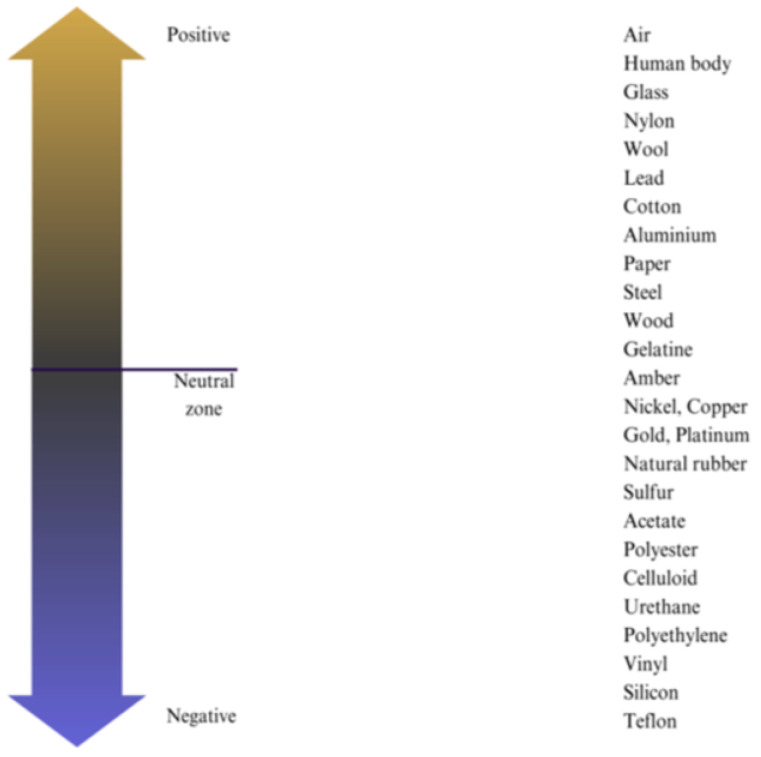
Triboelectric series shows the comparative tendency of materials to either gain or lose electrons.

When two materials come into contact, one substance becomes positively charged while the other material becomes negatively charged. Greater separation between particles results in a higher electrical charge transfer, while if they remain close, it leads to less or no charge transfer [3,52]. Figure 3 shows the triboelectric series, which classifies materials according to their propensity to gain or lose electrons.

**Figure 4:**
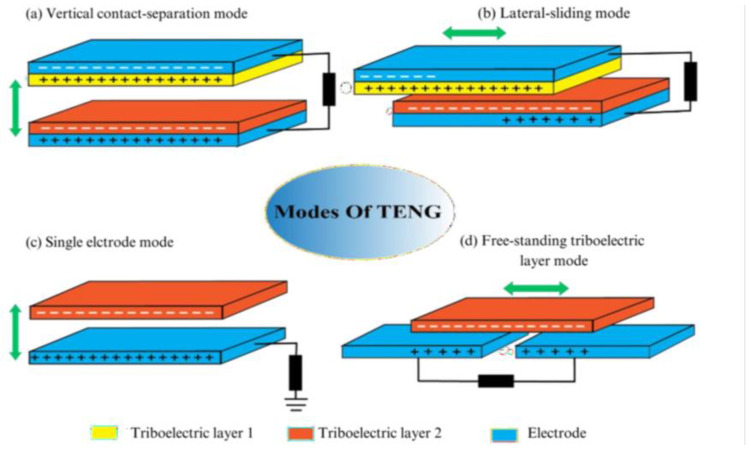
The four fundamental working modes of TENG. (**a**) The vertical contact–separation mode. (**b**) The lateral sliding mode. (**c**) The single electrode mode. (**d**) The free-standing mode.

TENGs offer a promising method for generating electricity independently and sustainably, reducing reliance on conventional batteries and driving the development of more efficient and eco-friendly electronic devices. TENGs offer diverse possibilities for converting mechanical energy into electrical power, with applications ranging from large-scale to small-scale energy harvesting and self-powered sensors [53,54]. Triboelectric nanogenerators can be used for various applications such as macro or microscale energy harvesting and self-powered sensors by converting mechanical energy into electricity. New designs are being developed to harness energy through the process of tribo-electrifying structural dielectrics or freely available ambient triboelectric sources. The designs are primarily categorized into contact-separation mode, sliding mode, single electrode mode, and free-standing triboelectric layer mode [55,56,57]. Figure 4 shows the four basic modes of triboelectric nanogenerators, including the vertical contact–separation mode, the lateral sliding mode, the single electrode mode, and the free-standing mode.

Improving the effectiveness of wearable TENGs is a challenging task that requires addressing several crucial issues [58,59]. Self-discharge in a triboelectric nanogenerator (TENG) refers to the gradual dissipation of electrical charge due to internal leakage [59,60]. The operational efficiency of a triboelectric nanogenerator (TENG) is significantly affected by self-discharge, which decreases the amount of electrical energy that can be extracted from the device. Interface optimization is crucial for enhancing the flow of electric charge across materials by ensuring they have unique triboelectric characteristics. However, selecting the optimal combination of materials is a difficult task as the performance of TENGs is highly dependent on the specific materials utilized [18,57,61]. This research article examines the several factors involved in embroidery and how they affect the performance of nanogenerators, specifically in the case of ETENGs. Our goal is to examine the effect of the embroidery parameters on the energy conversion in ETENGs. Through this investigation, we aim to discover valuable knowledge that not only improves our understanding of the fundamental processes controlling ETENG operation but also facilitates the creation of more efficient and adaptable energy harvesting nanogenerators based on embroidery.

## 2. Materials and Methods

This study introduces a method for manufacturing triboelectric nanogenerators by using embroidery techniques to incorporate conductive substrates with polyester and nylon 66 yarns. This results in the development of flexible and wearable energy-harvesting nanogenerators with various applications. Embroidery allows for precise control of thread length and line spacing, facilitating a methodical examination of their impact on TENG performance. By controlling these factors, our objective is to optimize the design of the triboelectric nanogenerator (TENG) in order to achieve improved power generation. The study also investigates the use of tapping and sliding devices to measure the electrical outputs of embroidered triboelectric nanogenerators, offering vital insights into their practical use in energy harvesting applications.

We created embroidered energy-harvesting nanogenerators utilizing the triboelectric effect, using 100% polyester yarn embroidered on Shieldex^®^ Medtex P130 conductive fabric manufactured by Shieldex, Bremen, Germany, and were evaluated against a nylon 66 triboelectric layered sample. The embroidered triboelectric nanogenerator converts mechanical energy generated by friction and tapping motion into electrical power through the triboelectric effect and electrostatic induction principles.

The polyester yarn is tribo-negative, and the nylon 66 yarn is tribo-positive. Nine different samples were made using various combinations of stitch lengths and line spacings, identified as ETFS1.1 through ETFS3.3, as shown in Table 1. Specialized devices, as shown in were used to conduct frictional and tapping tests in order to assess electrical outputs, such as short circuit current, open circuit voltage, and capacitor charging. The frictional device, which is controlled by a DC motor and reciprocating mechanism, allows the triboelectric nanogenerator to function in both lateral sliding mode and free-standing mode. The speed, reciprocating length, and frequencies of the nanogenerator can be adjusted as required to obtain output. The tapping device has features to perform in both the vertical contact–separation mode and the single electrode mode. It provides options for controlling the applied pressure, frequency, and contact time of the TENG samples. Current measurements were also made at different pressures to evaluate the influence of pressure on the performance of the Triboelectric Nanogenerator (TENG).

**Figure 5:**
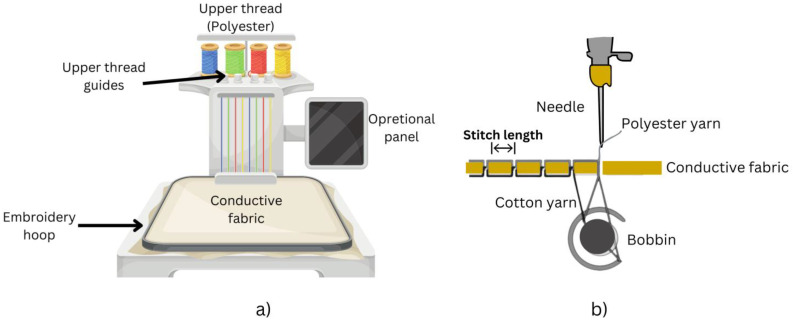
Schematic diagram of the (**a**) embroidery machine and (**b**) embroidery.

Choosing the right materials and embroidery parameters is crucial for generating static electricity effectively. An optimal combination of yarns can greatly enhance overall performance. Surface configuration and texture of materials are important for good surface structure. The main samples use polyester yarn. Polyester is a tribo-negative material, having surface electrons during tapping and friction. We utilized a 100% polyester yarn certified by STANDARD 100 by OEKO-TEX® [62] and made by Madeira, Freiburg, Germany. Nylon 66 was chosen as a tribo-positive sample for its strong electron-donating capabilities. Figure 5 shows the schematic diagram of the embroidery machine and embroidery.

**Figure 6:**
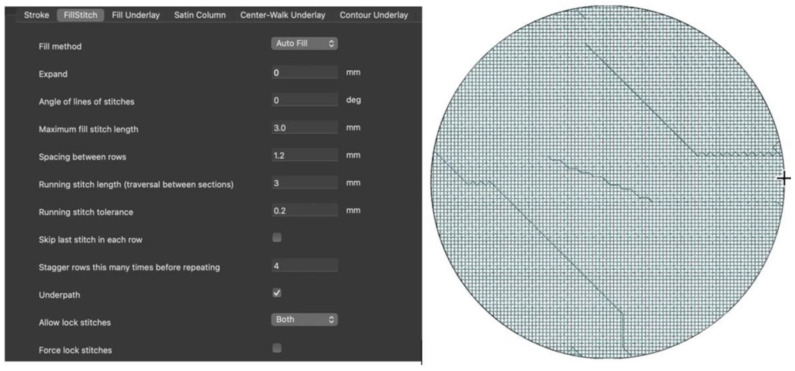
Different parameters to control the surface morphology of ETENG, Inkstitch v3.0.1 (an extension of Inkscape software).

**Figure 7:**
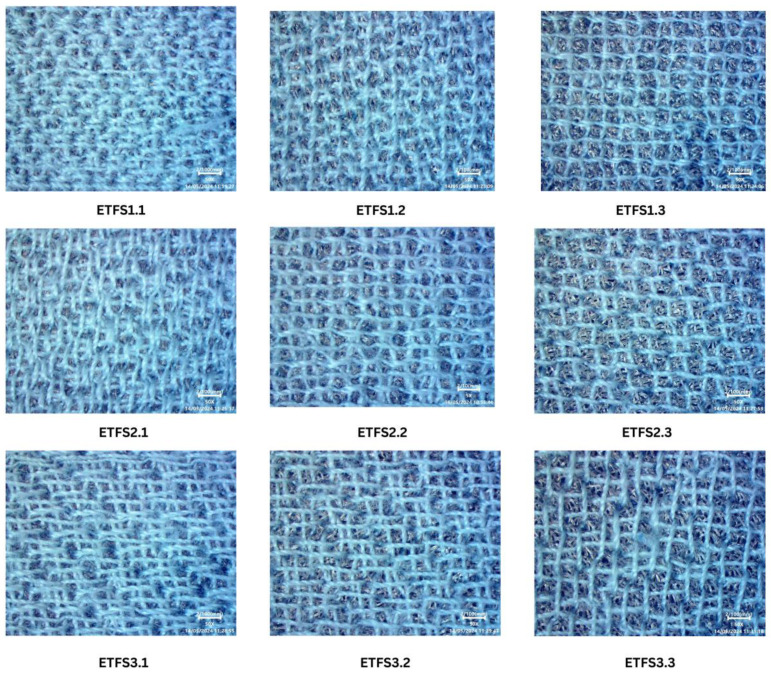
Surface morphology of ETENG.

Conductive cloth is the ideal choice for bending and breathable electrodes in portable TENG devices. It functions as a terminal and electron collector for the embroidery triboelectric nanogenerator so that TENG efficiently gathers charges during sliding and tapping motions. We utilized Shieldex^®^ Med-tex P130 manufactured by Shieldex, Bremen, Germany conductive cloth as the electrode for creating the embroidered TENG sample. The Shieldex^®^ Medtex P130 fabric incorporates silver metallization into its unique Stretch-Tricot knitted design. The fabric is made up of 78% polyamide and 22% elastane, providing remarkable flexibility in both the lengthwise and crosswise threads. The abrasion resistance is roughly ≤9000 cycles, and the combination of materials provides outstanding flexibility and comfort. Containing about 20% silver, Shieldex^®^ Med-tex P130 is ideal for wearable devices due to its conductive and antimicrobial characteristics. The fabric with the STANDARD 100 by OEKO-TEX® certification is both safe and sustainable. The product ensures ecological responsibility by achieving these standards. The Brother PR670E embroidery machine (Manufactured by Brother Industries, Ltd., Nagoya, Japan) is used to generate effective TENG samples on the conductive materials and detailed patterns on cloth. The Brother PR670E is a good option for making a precise and versatile embroidery TENG sample because of its user-friendly features. Figure 6 shows the different parameters with the simulation window of the Inkstitch that is used to control the line spacing and stitch length in ETENG preparation. The surface morphology of ETENG is shown in Figure 7.

**Figure 8:**
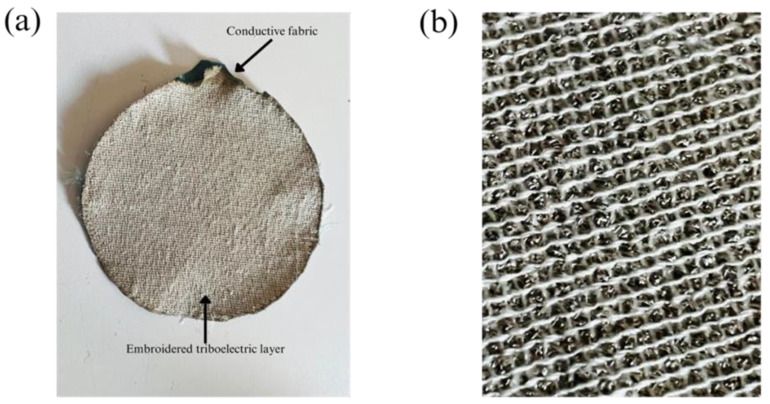
(**a**) The main sample made by 100% Polyester yarn embroidering on conductive fabric, (**b**) magnified view.

Developing and evaluating triboelectric nano-generators depends significantly on sample design, particularly when aiming for advanced features such as practicality, flexibility, and reduced weight. We utilized Ink/Stitch, an extension of Inkscape v 1.3, to create circular shapes with fill stitch embroidered TENG with different stitch lengths and line spacing factors. The fill stitch consists of underlay and overlay layers oriented perpendicular to each other. Figure 8a,b shows the main sample made of 100% Polyester yarn embroidering on conductive fabric, and magnified view of the fabric respectively.

**Figure 9:**
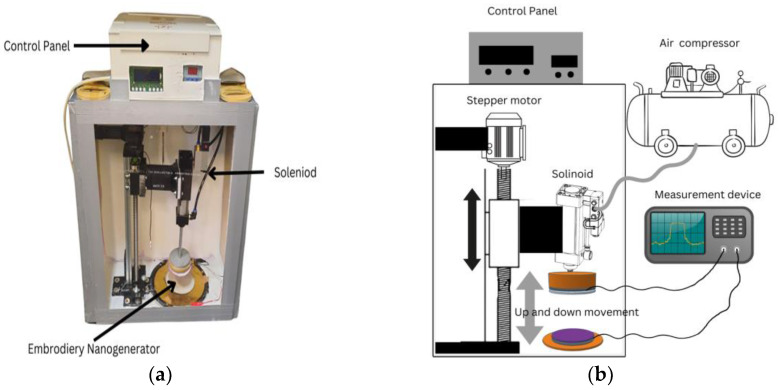
(**a**) Tapping characterization device used to characterize ETENG (**b**) Schematic diagram of the tapping device.

The polyester yarn was used in the embroidery machine to create the triboelectric layer, while the cotton yarn was used for the bobbin thread at the back of the fabric. The pliable conductive material was accurately trimmed to fit the dimensions of the embroidery hoop and connected to a fixed fabric in the hoop. A thin woven fabric was placed beneath the conductive fabric to serve as a basis and avoid shrinkage during the embroidery procedures. The embroidery frame’s four magnetic clamps guaranteed that the fabrics were uniformly and smoothly stretched, resulting in an optimum output. The fill stitch sample created in Inkscape software was then loaded into the machine to begin the embroidery process. Similarly, the tribo-positive sample was created using the nylon 66 yarn embroidered on the conductive substrate. The nylon 66 has a stitch length of 2 mm and a line spacing of 1 mm. The line spacing was minimized to ensure complete coverage of the electrode and prevent system short circuits caused by contact between electrodes of tribo-positive and tribo-negative samples during testing. Figure 9a,b shows the specialized tapping device and its schematic diagram respectively, used to characterize the output of ETENG.

The tapping test process was conducted using a tapping device, and the results were precisely measured with a Keithley 610c electrometer (manufactured by Keithley Instruments, Solon, OH, USA). The embroidered TENG needs to be directly integrated with the electrometer for accurate evaluation. The polyester sample was securely attached to the air-operated moving foot of the device, while the nylon 66 sample was positioned 50 mm below to guarantee precise separation. The tapping test maintained a constant tapping frequency of 2 Hz, applying a force (502 N) of one bar across an area of the main sample of 50.27 cm^2^.

**Figure 10:**
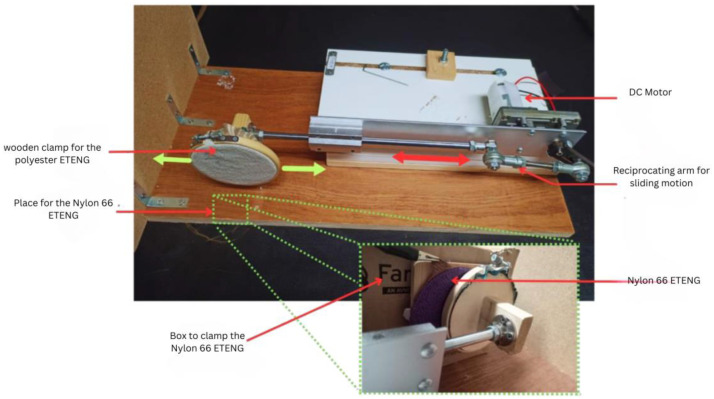
Sliding test device setup for characterization of the embroidery TENG.

Another specially developed device, shown in Figure 10, conducted the friction test by utilizing lateral sliding motion powered by a DC motor. The mechanical arm has a clamp to secure the main sample, enabling horizontal movement at different frequencies up to 2 Hz. A portable rigid box is used to secure the nylon 66 sample, ensuring consistent contact with the mechanical arm to provide friction during machine operation.

The machine frame and sample mounting clamps are constructed from wood with a diameter of 10 cm. The wooden construction is ideal for electrostatic testing as it has a negligible impact on test results. Once the polyester and nylon 66 samples were secured in the machine, a connection to the circuit or measuring device was made. In Figure 10, the main sample is made of polyester, while the reference sample is made of nylon 66.

We conducted current measurements on our samples using a tapping and sliding characterization machine for 60 s, repeating the process three times for each sample. We measured open-circuit voltage, short-circuit current, and induced surface charge using an electrometer and current measurement circuit sequentially. The tapping tests were performed with a 50 mm gap between two triboelectric samples and 1 bar pressure on a 201 cm^2^ active area of the main sample made of 100% polyester. The sliding tests were conducted at a frequency of 1.5 Hz.

A full-wave rectifier circuit was used to analyze charge storage in various capacitors by tapping and sliding tests. Developing circuits that can convert AC voltages into useful DC power sources is crucial for maximizing the potential of TENG. We utilized a rectifier diode to convert the AC voltage generated by the TENG into DC voltage, which was then stored in a capacitor.

**Figure 11:**
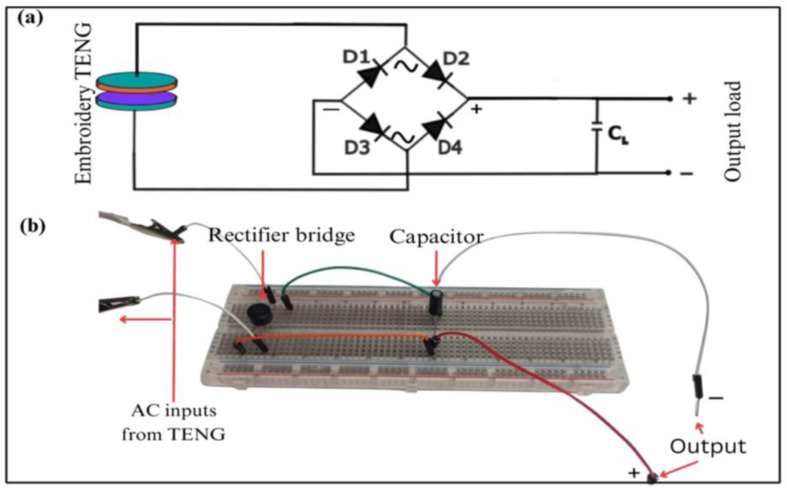
(**a**) Schematic diagram of a rectifier circuit for energy harvesting in a capacitor, (**b**) rectifier circuit with capacitor.

The TENG’s output was connected to the input of the rectifier diode, and then the output of the rectifier diode was connected to the positive terminal of the capacitor. The negative terminal of the capacitor is linked to the negative lead of the rectifier. Figure 11a,b shows the schematic diagram of a rectifier circuit for energy harvesting in a capacitor, and rectifier circuit with capacitor respectively.

The top-performing sample was tested with 1 μF, 4.7 μF, and 10 μF capacitors to determine the most suitable one for the initial step of power storage in the embroidered energy harvester.

## 3. Results

**Figure 12:**
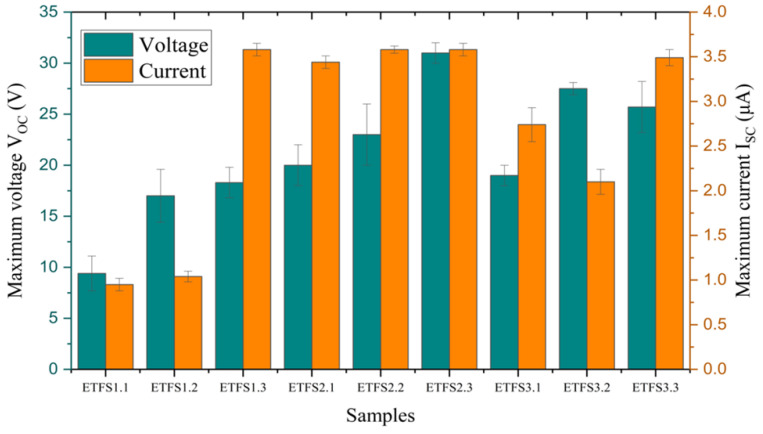
Experimental results of tapping test average maximum open circuit voltage and average maximum short circuits current.

**Figure 13:**
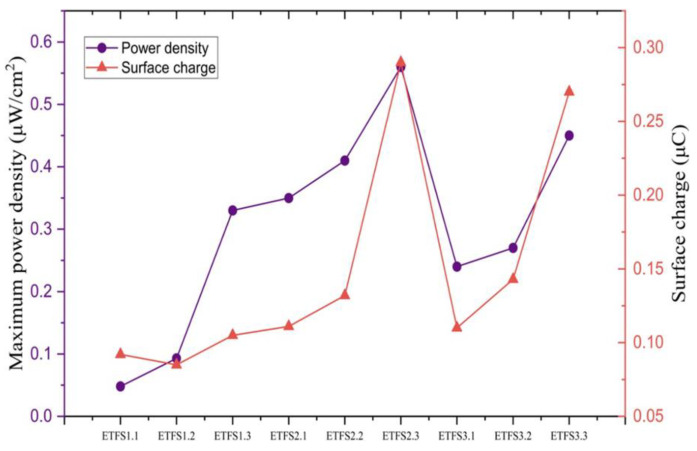
Maximum power density and surface charge generated in 60 s by tapping test in 2 Hz frequency.

Figure 12 displays the average maximum open-circuit voltage, and average maximum short-circuit current for a tapping test conducted with 1 bar machine pressure, 201 cm^2^ active sample area, and a 50 mm spacing between two triboelectric layers, as recorded by the electrometer. ETFS2.3 and ETFS3.2 are optimal samples for producing high voltage during tapping tests. We analyzed the samples further to obtain the highest power density vs surface charge (Figure 13).

Based on Figure 13, it can be observed that both power density and surface charge for the tapping test exhibit an upward trend as the line spacing increases. This trend is consistent across all three groups: ETFS1, ETFS2, and ETFS3. The ETFS2.3 and ETFS3.3 samples, with line spacing of 1.2 mm and stitch lengths of 2 mm and 3 mm, respectively, performed better than samples with a shorter line spacing.

Based on the results, we can conclude that ETFS 2.3 demonstrates the highest performance in terms of open-circuit voltage, short-circuit current, induced surface charge, and power density, making it the top performer on average, indicating that the 2 mm stitch length is close to the optimum for a line spacing of 1.2 mm.

**Figure 14:**
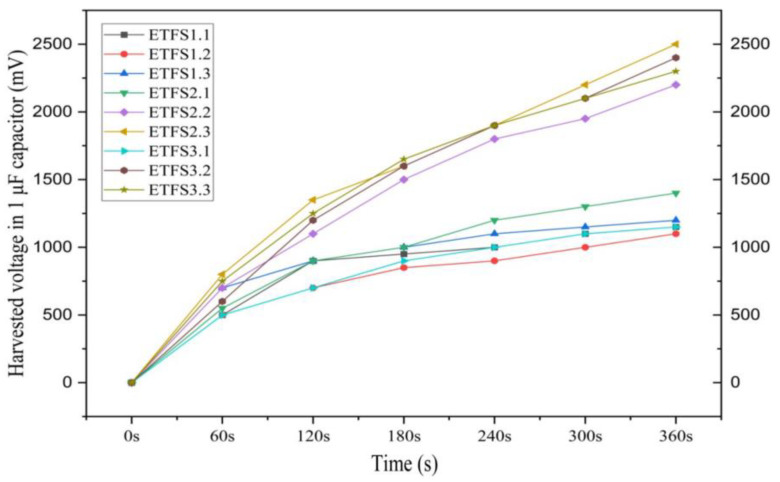
The harvested voltage in 1 μF capacitor during the tapping test.

**Figure 15:**
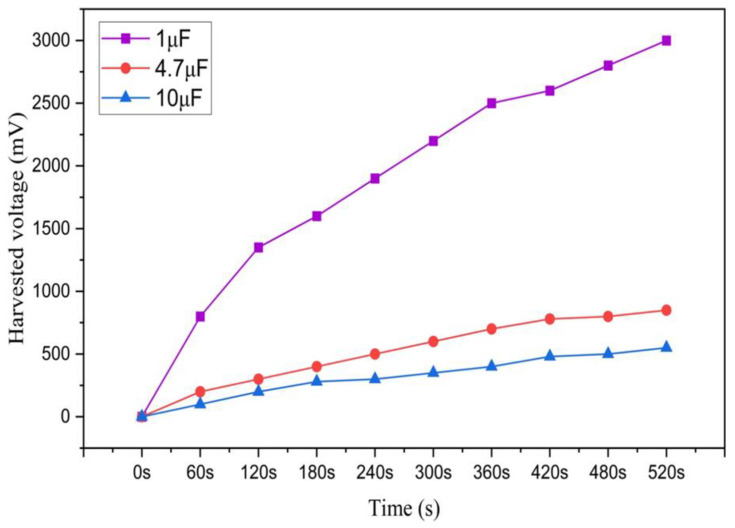
The harvested voltage of sample ETFS2.3 in 1 μF capacitor for tapping test throughout 520 s.

Next, the harvested voltage was determined for all the samples, see Figure 14. It can be concluded that ETFS2.3 is also the most effective sample for conducting tapping tests of power density and energy harvesting in a 1 μF capacitor with a force of 502 N at 1 bar pressure and a frequency of 2 hertz applied to the devices during the capacitor charging experiments. Figure 15 shows the harvested voltage of sample ETFS2.3 in 1 μF capacitor for tapping test.

**Figure 16:**
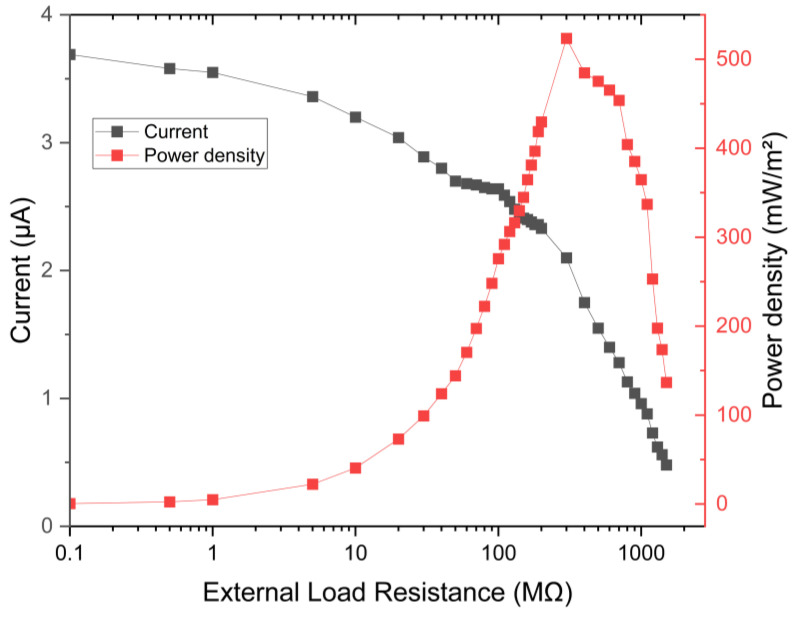
External load resistance to obtain the maximum power density.

In order to conduct a more thorough investigation of our best energy harvesting sample ETFS2.3, we incorporated two additional capacitors, one with a capacitance of 4.7 μF and another with a capacitance of 10 μF. These capacitors were utilized to store the collected energy in a capacitor through a rectifier circuit. The 1 µF capacitor stored a maximum energy of 4.5 millijoules (mJ) for a duration of 520 s, resulting in a voltage of 3000 mV. The harvested energy was sufficient to completely illuminate a few LEDs, demonstrating the potential for energy usage on a smaller scale. The optimal load resistance to achieve the maximum power density in an ETENG is 300 MΩ, as shown in Figure 16.

### 3.1. Sliding Test Result

**Figure 17:**
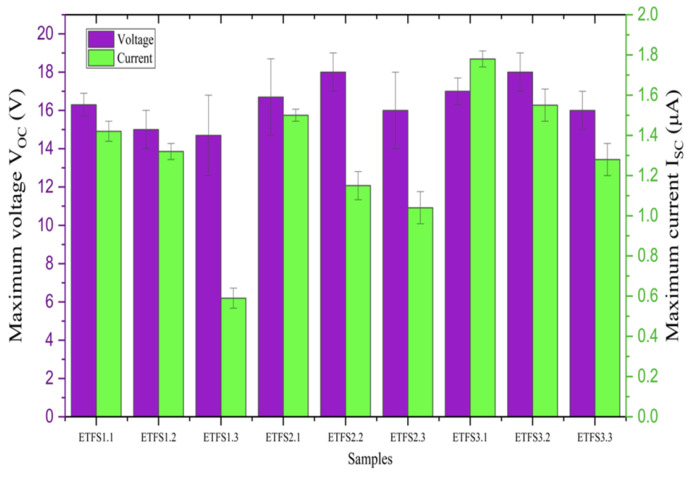
Experimental results of average maximum open circuit voltage and average maximum short circuits current for the sliding test.

**Figure 18:**
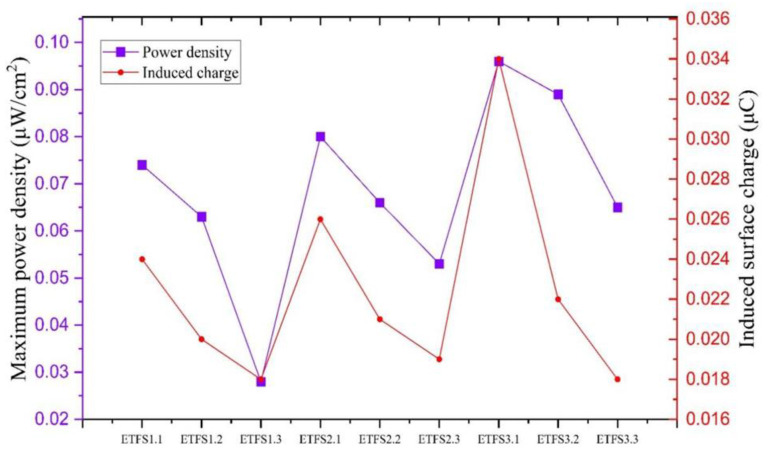
Maximum power density and surface charge generated in 60 s for sliding test.

Next, we present the sliding test results in Figure 17. The data indicate that ETFS2.2, ETFS3.1, and ETFS3.2 are the most effective in generating voltage. However, ETFS3.1 stands out as the most consistent performance compared to ETFS2.2 and ETFS3.2. However, the top three options have a greater stitch length and a smaller line spacing: a different result from the tapping test. The power density and induced surface charge are given in Figure 18. A downward trend is now clear for all sample types (ETSF1,2 and 3) with increasing line spacing.

**Figure 19:**
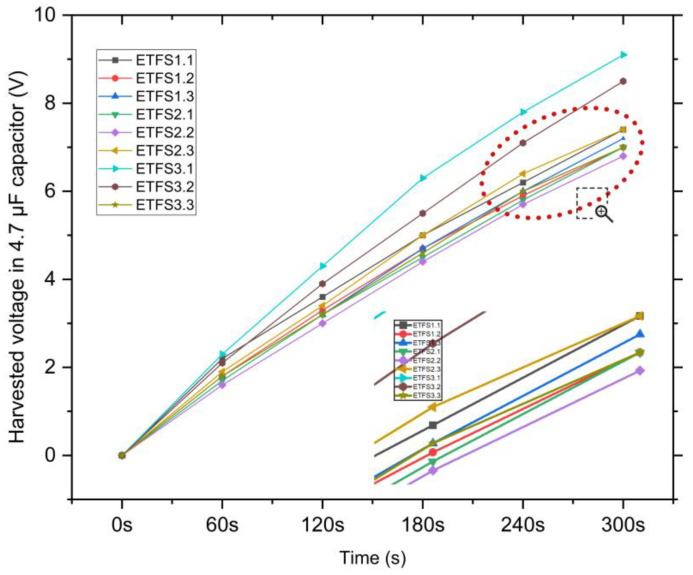
Harvested voltage in 4.7 μF capacitor by sliding at 1.5 Hz frequency.

**Figure 20:**
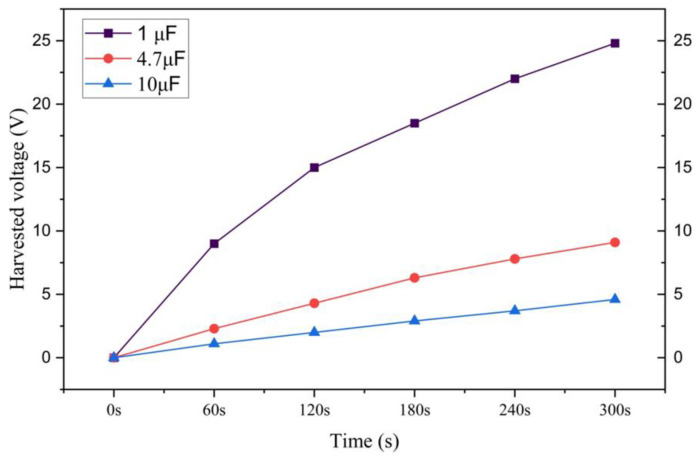
The harvested voltage of sample ETFS3.1 in different capacitance capacitors.

Figure 19 shows the outcome of energy harvesting using a full wave rectifier bridge circuit on a 4.7 μF capacitor during a lateral sliding experiment. The y-axis displays the voltage (in volts), while the x-axis is time duration. Out of all the samples in this experiment, ETFS3.1 produces the highest energy outputs. In order to gather further results, a subsequent test was carried out using capacitors of 1 μF, 4.7 μF, and 10 μF for ETFS3.1, see Figure 20.

**Figure 21:**
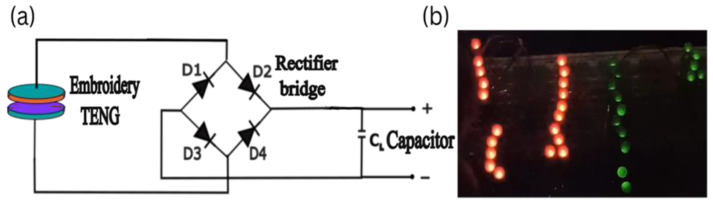
(**a**) Rectifier circuit for energy harvesting in capacitor, (**b**) lighted LEDs by harvested energy.

It was possible to charge a capacitor of 1 μF with a voltage of 24.8 V over a period of 300 s. We utilized the captured energy to illuminate a total of 20 small red LEDs, together with 11 green LEDs, as shown in Figure 21.

Based on this experiment, it is evident that a 1 μF capacitor is the most effective in storing harvested energy through the rectifier. Based on the comprehensive analysis of the findings, it is evident that the tapping test consistently yields the greatest values for both the maximum open-circuit voltage and short-circuit current. This is observed during testing of contact and separation between two triboelectric layers, with a distance of 50 mm between them and a pressure of 1 bar applied to an active sample area of 201 cm^2^. The friction test, on the other hand, reveals a somewhat lower instantaneous maximum open-circuit voltage and short-circuit current compared to the tapping test. During the tapping tests, samples that had larger interline spacing and lesser quantities of triboelectric yarn demonstrated improved power density performance. The ETFS2.3 sample, including a stitch length of 3 mm and a line spacing of 1.2 mm, exhibited the best performance.

However, during frictional tests, sample ETFS3.1 demonstrated superior performance compared to the other samples, suggesting that stitch length has an impact on the generation of electricity through friction, with longer stitch length and smaller line spacing as beneficial.

**Figure 22:**
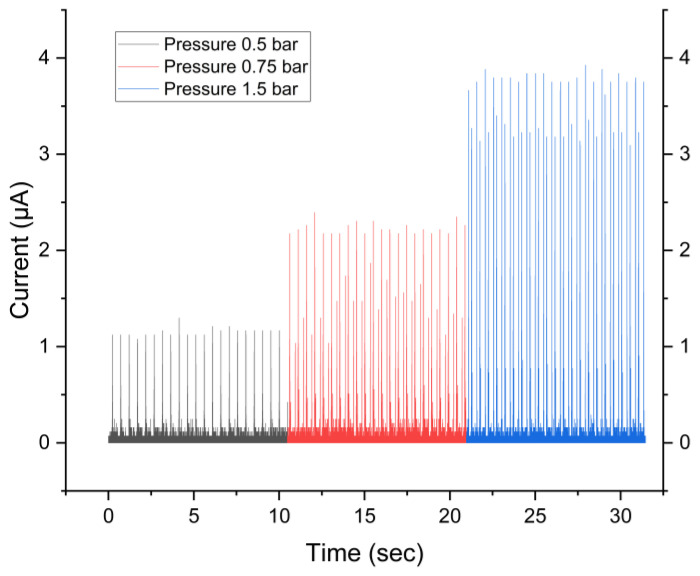
Measurement of the short current at different pressures.

Measurement of the current is performed at different pressures to check the effect of pressure and validate the different options of the triboelectric/electrostatic characterization device, as shown in Figure 22. Measuring the current under different pressure settings is an important part of evaluating the performance of ETENGs and understanding how pressure affects energy generation. Through a systematic changing of the pressure exerted on the ETENG, it can be determined the effects of mechanical forces on the electrical signal produced. This investigation provides insights into the triboelectric device’s responsiveness to external stimuli and its potential for practical use in situations where pressure fluctuations are frequent. Moreover, this experiment validates the effectiveness of several features of the triboelectric and electrostatic characterization equipment used to evaluate the performance of ETENGs. As can be seen from Figure 22, higher pressure during tapping leads to a higher short circuit current. The reason for this is that increasing the pressure increases the contact area and friction between the embroidery TENG, leading to a higher transfer of charges and, consequently, higher short-current.

### 3.2. Practical Integration

**Figure 23:**
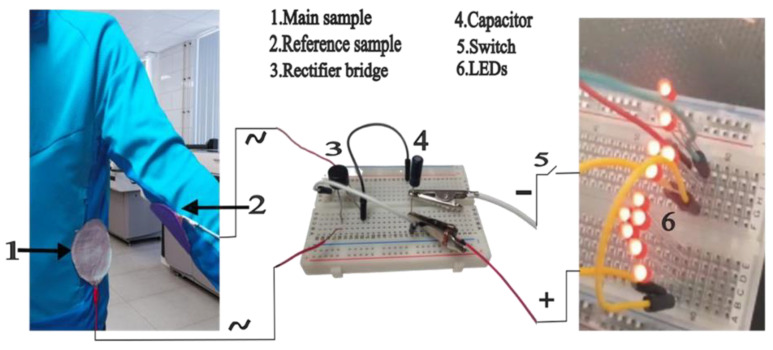
Energy harvesting from human kinetics by generating friction on the side torso.

The suggested embroidery energy harvesting nanogenerator is seamlessly stitched into traditional textile apparel due to the exclusive use of textile materials in all the components of the TENG. We positioned our embroidery triboelectric nanogenerator on the bottom regions of a jacket’s body. The most high-performing polyester sample, ETFS3.1, was attached to the lower torso, whereas the nylon 66 reference sample was placed on the forearm (see Figure 23). During ambulation or locomotion, two triboelectric layers make contact due to inherent hand motions. Thus, the process of contact separation and sliding between the components generates energy that is stored in the capacitor. The prototype was subjected to testing on the human body within a controlled electrostatic environment, specifically to replicate walking motion. The outcome of the test was the successful generation of 12 volts of electricity, which was stored in a 1 µF capacitor, resulting in a charge of 12 microcoulombs, achieved in a remarkably short time of only 120 s. Through the process of energy collecting, it is possible to fully illuminate a total of 11 red LEDs.

### 3.3. Washability

The best-performing ETENG samples undergo washability tests in a commercial washing machine (model ES-NB814WNA, Sharp, Osaka, Japan), complying with the instructions of ISO 6330:2021 (Textiles—Domestic washing and drying procedures for textile testing) [63]. The detergent used in the tests consisted of a mixture of 15.4 g ECE detergent, 4 g perborate, and 0.6 g bleach activator. The electrical output of ETENG samples was tested prior to and after different washing durations (10, 20, and 30 h) in order to evaluate changes in output performance.

**Figure 24:**
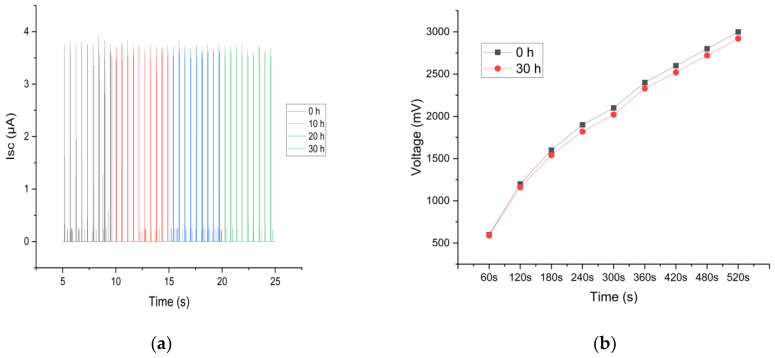
Output performance after washing: (**a**) short circuit current, (**b**) capacitor charging of 1 μF.

We measured the short-circuit current (Isc), open-circuit voltage (Vo), and charging capability with a 1 μF capacitor. The results, as shown in Figure 24, indicated that ETENG exhibited a slight decrease in output after washing. The short-circuit current and open-circuit voltage are given below:

- At 0 h, the output was 3.66 µA and 32.17 V.
- After 10 h of washing, the output was 3.62 µA and 32.07 V.
- After 20 h of washing, the output was 3.60 µA and 31.59 V.
- After 30 h of washing, the output was 3.56 µA and 30.94 V.

**Figure 25:**
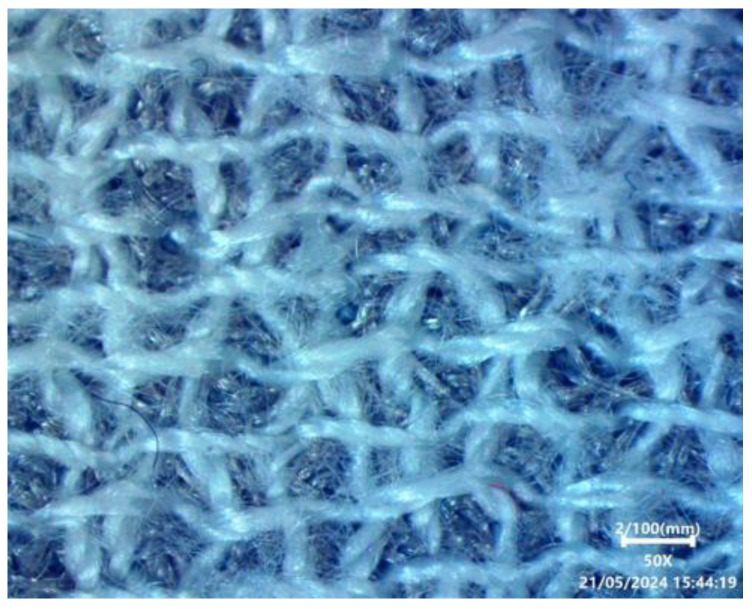
Optical microscope image of ETENG after 30 h of washing.

These results indicate that ETENG has strong mechanical durability and maintains a major portion of its electrical output even after heavy washing. Figure 25 shows the optical microscope image of ETENG after 30 h of washing.

### 3.4. Durability

**Figure 26:**
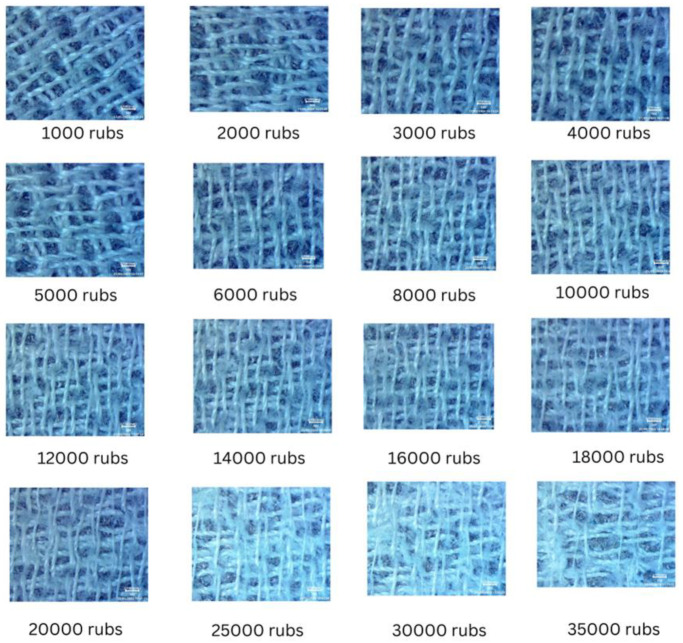
Martindale test for the mechanical durability of ETENG.

Martindale test was carried out to evaluate the durability of ETENG against mechanical friction. This test is to evaluate the mechanical durability of textile materials when they undergo repetitive friction. Figure 26 shows the results, which clearly indicate the high mechanical stability of ETENG. During the test, the ETENG showed two thread breakages between 30,000 and 35,000 rub times under the force of 12 kPa.

## 4. Discussion

The observed variations in TENG performance emphasize the importance of stitch length and line spacing factors in energy generation. Increased line spacing in the tribo-negative component confirms effective charge separation, leading to improved power output under tapping. Under sliding, however, decreasing line space leads to improved power output, making it important to tune the line spacing based on the application. Stitch length is another critical factor in generating energy through triboelectrification, and longer stitches provide better performance under sliding while tapping showed an optimum with a stitch length of 2 mm. These findings highlight the significance of fine-tuning embroidery parameters for TENG applications. The displayed energy harvesting abilities further confirm the practicality of ETENG for wearable and portable electronic devices. The utilization of larger embroidery stitches and close line spacing results in a higher surface area when compared with shorter stitches and wider line spacing. However, when there is an optimal distance between two lines, it results in an increase in both surface area and surface roughness.

During the tapping test, we noticed that samples with greater line spacing and a smaller amount of triboelectric yarn had superior performance in terms of power density. The most high-performing sample is ETFS2.3, which consists of a stitch length of 2 mm and a line spacing of 1.2 mm. However, when there is friction, stitches with a greater length can provide more effective friction compared to stitches with a shorter length. Furthermore, samples with a greater stitch density and smaller spacing had a larger quantity of triboelectric yarn, allowing for the generation of a greater amount of electrical charge. The stitch length is adjusted to maximize power generation. During our experimentation, we found that the ETENG had greater energy harvesting ability in a 1 μF capacitor during friction tests as compared to tapping tests, though the maximum voltage and current were lower. The reason for this is the constant presence of friction, whereas in tapping there are interruptions due to contacts and separations. Optimizing the thread length during friction is crucial for achieving higher output. This is because using a longer stitch length during the rubbing of samples results in improved friction and a greater number of contact points, leading to increased power production.

## 5. Conclusions

This study conducts a thorough examination of how embroidery factors affect the output performance of triboelectric nanogenerators. Through a methodical change of stitch length and line spacing, we have determined the most effective configurations for maximizing power generation. The ETFS2.3 sample, which had a stitch length of 2 mm and a line spacing of 1.2 mm, demonstrated maximum power density performance for the tapping test. The energy harvesting prototype provided additional evidence of the potential of embroidered triboelectric nanogenerators for practical use in wearables. In summary, this research offers useful knowledge regarding the development and enhancement of embroidered triboelectric nanogenerator output for the purpose of sustainable energy generation. This research has examined the application of an energy-harvesting embroidered sensor that utilizes the triboelectric effect, employing 100% polyester and nylon 66 triboelectric yarns. Concentrating on thoroughly assessing the electrical output efficiency while tapping and sliding. Furthermore, this research has specifically concentrated on creating embroidered TENG and refining the manufacturing parameters. It has also examined how modifications in these factors impact the electrical output performance of the TENG.

We were able to effectively extract energy from embroidered TENG using both specialized tapping and sliding devices, as well as from the human kinematics during the slow running. We obtained a maximum of 307.5 μJ (24.8 V) of energy from a 1 μF capacitor by a sliding motion with a frequency of 1.5 Hz over a period of 300 s. Additionally, we extracted 72 μJ (12 V) of energy from a 1 μF capacitor in 120 s using human motion with the ETFS3.1 sample. We obtained a maximum energy of 4.5 μJ (3 V) by using a tapping device to charge a 1 μF capacitor. This energy was harvested from the ETFS2.3, which was subjected to a tapping motion at a frequency of 2 Hz and a separation distance of 50 mm. The experiment’s precise combinations of stitch length and line spacing resulted in an increase in maximum voltage, maximum current, and power output. It was noted that samples with medium stitch lengths and greater line spacing exhibited a higher level of triboelectric charging during contact and separation. We noted a distinct pattern for the sliding mode. A sample with a relatively longer stitch length and shorter line spacing produces a greater amount of electric output. The reason is that an optimal comparative increase in stitch length allows for a larger contact area during sliding motion.

## Figures and Tables

**Table 1:** Specification for designing the polyester samples.

Sample Name	Stitch Length *l* (mm)	Spacing between Lines *d* (mm)
ETFS1.1	1.5	0.65
ETFS1.2	1.5	0.90
ETFS1.3	1.5	1.2
ETFS2.1	2	0.65
ETFS2.2	2	0.90
ETFS2.3	2	1.2
ETFS3.1	3	0.65
ETFS3.2	3	0.90
ETFS3.3	3	1.2

## Data Availability

The data presented in this study are available on request from the corresponding author.
